# Navigation performance in glaucoma: virtual-reality-based assessment of path integration

**DOI:** 10.1038/s41598-024-72040-8

**Published:** 2024-09-12

**Authors:** Safa Andac, Francie H. Stolle, Matthieu Bernard, Khaldoon O. Al-Nosairy, Thomas Wolbers, Michael B. Hoffmann

**Affiliations:** 1 Ophthalmic Department, Section for Clinical and Experimental Sensory Physiology, Otto-Von-Guericke University Magdeburg, Leipziger Str. 44, 39120 Magdeburg, Germany; 2 German Center for Neurodegenerative Diseases, Magdeburg, Germany; 3 Center for Behavioral Brain Sciences, Magdeburg, Germany

**Keywords:** Navigation, Path integration, Visual field, Glaucoma, Virtual reality, Visual system, Optic nerve diseases, Neurodegeneration

## Abstract

Navigation is essential for moving between locations in our daily lives. We investigated the relationship between visual impairment in glaucoma and path-integration-based navigation. Fourteen glaucoma and 15 controls underwent ophthalmological examination (including visual acuity (logMAR), visual field sensitivity (MD: mean deviation from matched reference cohort), and peripapillary retinal nerve fiber layer (pRNFL)). Both groups navigated physically in virtual reality (VR) environments during daylight and dawn conditions. Briefly, the participants traversed a path marked by three targets, subsequently pointing back to the path’s origin. Outcome measures included (i) travel-time, (ii) pointing-time, and (iii) Euclidian-distance error between indicated and starting position. Robust linear regression was conducted between visual function outcomes of the better eye and VR outcome measures. Glaucoma patients showed increase in travel-time (by 8.2 ± 1.7 s; *p* = 0.002) and in pointing-time (by 5.3 ± 1.6 s; *p* = 0.016). Predictors were MD for all outcome measures (*p* < 0.01) and pRNFL for travel-time (*p* < 0.01). The results suggest that the effect of glaucoma on the elapsed time depends on disease progression, i.e. people with stronger visual impairment need more time. This uncertainty during everyday navigation tasks may adversely affect their quality of life.

## Introduction

Glaucoma is an age-related neurodegenerative disease, which does not only lead to visual field deficits^1^, but also to changes in brain structure and function^2^, some of them related to visuo-motor coordination^3^. The impact of visual field loss on visuo-motor coordination might in turn affect postural control and orientation^3–5^, leading to an increase in incidence of falls^6–11^ and a decrease in mobility^12,13^. This highlights the need for surrogate glaucoma biomarkers related to spatial navigation^14^ as an integral part of mobility skills and eventually quality of life.

Spatial navigation employs wayfinding and path integration skills to maintain routes during locomotion with critical importance for many daily life activities. Previous studies report degraded navigation skills in elderly^15^ and visually impaired^16–18^. This highlights the relevance of visual input for spatial navigation. E.g., Daga et al^19^ reported worse performance of glaucoma patients during navigation in wayfinding tasks utilizing a virtual reality (VR) environment. In general, it appears that patients with visual field loss in the peripheral or central field have a reduced navigation performance in VR environments^20^ and that VR appears a valid tool to mirror and evaluate vision-related disabilities^21^. However, previous, so-called passive, VR-based navigation studies did not integrate relevant physical skills for navigation, such as mobility or active movement as in path integration. This is of importance, due to the relevance of external as well as body-based, e.g. vestibular or proprioceptive cues. Tests in real life situations might be an alternative and in fact indicate reduced performance in glaucoma^12,13^, but have limited experimental options. Therefore, integrating active movement during navigation in, so called active VR, holds specific potential in patient studies, due to its close relation to real-world situations. Moreover, active VR has the advantage of systematically controlled visual cues, less physical effort, being more engaging, and creating safer testing environments. This motivated us to investigate the effect of glaucoma on navigation, specifically path integration, with an active VR-setup. Path integration refers to the ability to keep track of one’s own position and orientation with respect to a reference-point. We conducted a path-integration navigation assessment in two different lighting conditions, i.e. daylight and dawn, with two participant groups, i.e. glaucoma or controls. The participants walked through an immersive VR environment and eventually pointed back to their origin. We assessed the performance in this path-integration task from the outcome measures, (i) travel time, (ii) pointing time, and (iii) distance error. As an effect of glaucoma (GLA), we hypothesized (i) the durations of travel time and pointing time to be prolonged and (ii) distance errors to be increased compared to control (C).

## Results

**Fig. 1:**
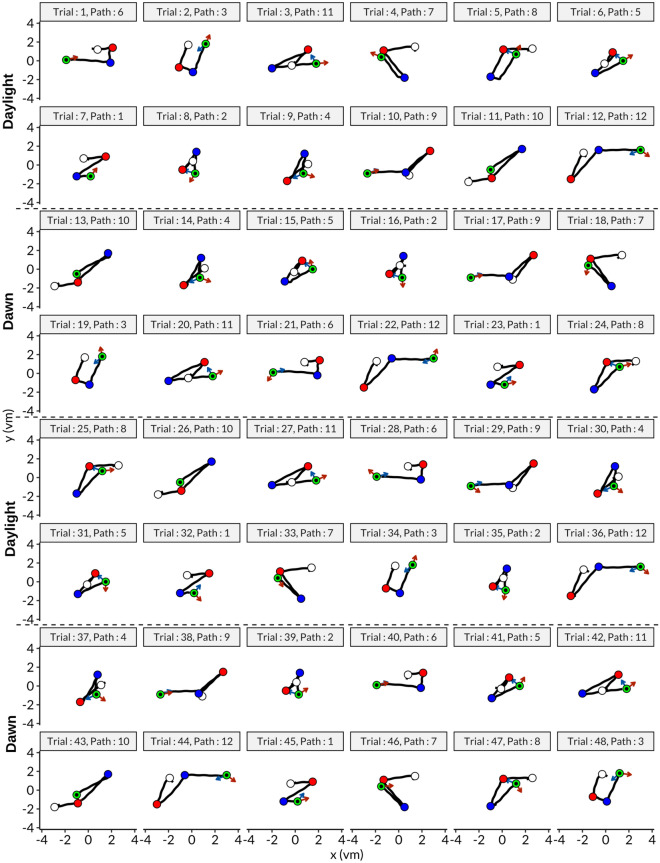
Movement data of a participant from GLA. In total there were 48 trials and one block had 12 trials, each block was repeated twice for both conditions, daylight and dawn, as indicated. Path numbers refer to same paths in Fig. 4D for convenience. Blue arrows indicate the expected direction whereas orange ones represent the response direction of the participant for pointing task. It is evident that movements of the participant did not follow exactly a straight line and also include head sway movements.

An overview the experimental data from a representative participant from the GLA group is given in Fig. 1. Here, the trajectories, pointing directions, and distance errors are depicted.

### Navigation performances

**Fig. 2:**
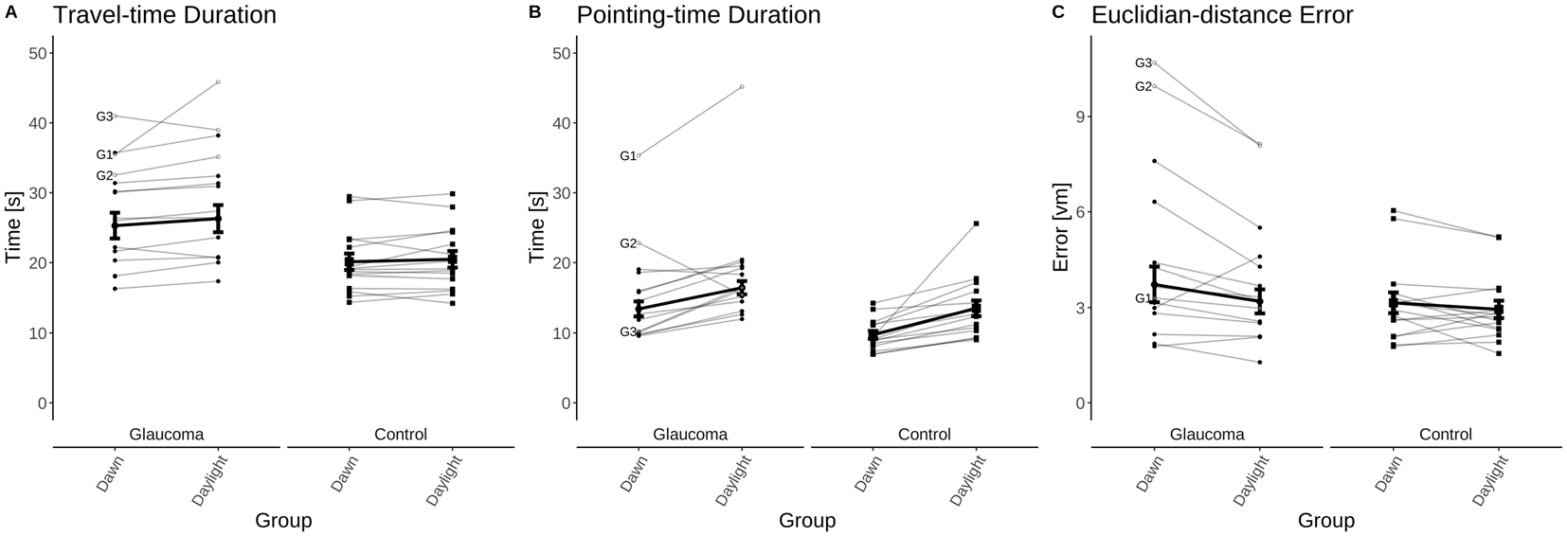
Mean ± standard error for the glaucoma (n = 14) and the control group (n = 15) (thick lines) and for the individuals (thin lines). The three glaucoma patients with severe visual field defects are highlighted (G1, G2, G3 as detailed in suppl. table 1).

We quantitatively assessed the performance of participants in the path-integration task using three different outcome measures, i.e. (i) travel-time, (ii) pointing-time, (iii) Euclidian-distance error (see Fig. 2):

(i) Travel-time (Fig. 2A): GLA moved slower, hence, spent more time than C (by 8.2 ± 2.3 s; GROUP effect; F(1,27) = 12.17; $$p<0.01$$). We also observed, independent of group, longer travel-time for daylight than for dawn (by 0.9 ± 0.4 s; LIGHTING effect; F(1,27) = 5.24; $$p=0.03$$). No interaction was found between the main effects LIGHTING and GROUP (F(1,27) = 2.11; $$p=0.16)$$.
(ii) Pointing-time (Fig. 2B): we found patterns similar to those for the travel-time measurement. GLA responded more slowly than C (by 5.3 ± 2.1 s; GROUP effect; F(1,27) = 6.67; $p=0.02$) and, independent of group, pointing-time was longer for daylight than for dawn (by 3.4 ± 0.7 s; LIGHTING effect; F(1,27) = 23.20; $p<0.001$). No interaction was found between the two main effects (F(1,27) = 0.31; $p=0.58$).
(iii) Euclidian-distance error (Fig. 2C): we did not observe any difference of the Euclidian-distance error between the two groups (no GROUP effect; F(1,27) = 2.95; $p=0.097$), while both groups’ performances were better at daylight (by 0.48 ± 0.17 vm; LIGHTING effect; F(1,27) = 8.54; $p<0.01$). Again, no interaction between the two main effects was evident (F(1,27) = 2.78; $p=0.11$).

In order to check whether our results were driven by three participants with severe visual field defects, we conducted the same analyses by excluding them in all three outcomes. We found the effects reported for the entire cohorts to persist (F(1,24) > 4.26; *p* < 0.05).

Ageing has an effect on navigation abilities^22^. While there was no significant age difference between GLA and C, we re-conducted the analyses with age as a covariate factor. As expected, the GROUP effect was still observed in time measures (F(1, 26) = 11.09; $$p<0.01$$ and F(1,26) = 5.21; $$p=0.03)$$. In contrast, the group-independent LIGHTING effect disappeared in all three outcome measures ($$p>0.05)$$, suggesting an age-dependence of the LIGHTING effect to be addressed in follow-up studies.

In summary, GLA were slower than C. Moreover, participants moved slower in daylight condition and they performed better in daylight condition in terms of finding the first checkpoint, but the effect was small and disappeared when age is considered.

### Relationship between navigation performances and visual impairments

**Fig. 3:**
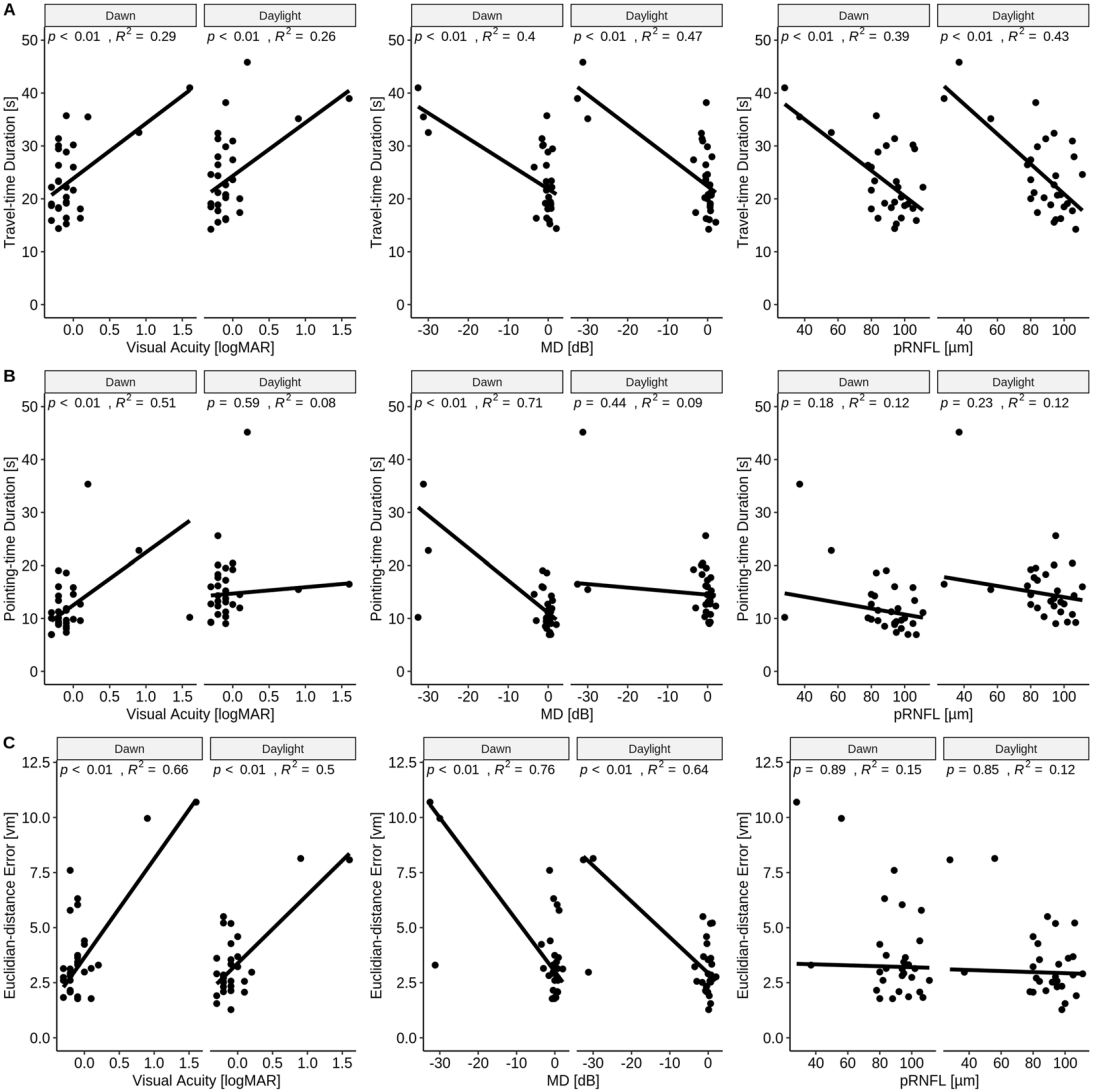
Robust regression analyses. The scatterplots illustrate the relation between visual impairment and outcome measures of path integration paradigm as detailed in the text.

In order to examine whether the above group effects, i.e. travel- and pointing-time, were actually associated with damage to the visual system in GLA, we assessed the relation of the above effects and parameters with ophthalmological measures. For that purpose, we applied robust linear regression analyses with bisquare weighting method^23^. An overview over all applied regressions (travel-time, pointing-time, distance error vs. VA, MD, pRNFL) is given in Fig. 3 and summarized below:

(i) Travel-time (Fig. 3A): Optimal predictors for travel-time were pRNFL and MD in both daylight and dawn conditions: An MD of 1 dB sensitivity loss corresponded to a travel-time delay of 0.58 s (95% CI 0.34–0.81; *p* < 0.001; ${R}^{2}=0.47$) for the daylight condition and of 0.48 s for dawn (95% CI 0.26–0.70; *p* < 0.001; ${R}^{2}=0.4$). A thinning of 1 µm in pRNFL corresponded to a travel-time delay of 0.28 s for daylight (95% CI 0.16–0.41; *p* < 0.001; ${R}^{2}=0.43$) and of 0.24 s for dawn (95% CI 0.13–0.36; *p* < 0.001; ${R}^{2}=0.39$).
(ii) Pointing-time (Fig. 3B): The best predictor for pointing-time was MD: 1 dB sensitivity loss corresponded to a pointing-time delay of 0.61 s (95% CI 0.46–0.76; *p* < 0.001; ${R}^{2}=0.71$) for dawn, while it was insignificant for daylight.
(iii) Euclidian-distance error (Fig. 3C): 1 dB sensitivity loss corresponded to an increase in distance error of 0.16 vm (95% CI 0.12–0.21; *p* < 0.001; ${R}^{2}=0.64$) for daylight and of 0.23 vm (95% CI 0.18–0.28; *p* < 0.001; ${R}^{2}=0.76$) for dawn.

## Discussion

In a VR-path-integration paradigm we found in accordance with our initial hypothesis (i) that GLA were slower than C in task completion as reflected by longer travel-time and pointing-time. In contrast to our initial hypothesis (ii) accuracy, i.e. distance error, was similar between groups. For the daylight condition, travel time of both participant groups was slower, while the performance was more accurate, but the group effect is negligible, as it was small and disappeared when age was included as a covariate. Remarkably, in GLA, both travel- and pointing-time correlated with the visual field damage (MD) and travel-time with pRNFL. Our findings suggest that travel time might be a potential biomarker for behaviorally relevant effects in glaucoma, even in early stages.

### Effect of glaucoma on duration of task completion

Previous research involved tasks with physical movement activities to comprehend how glaucoma affects people in daily life. They analyzed time to complete the task^13,14,24–26^ and spatio-temporal gait parameters^27–29^. In general, when glaucoma patients were instructed to complete a mobility task, e.g. timed-up and go test, dynamic gait index tests, a course with obstacles, they moved slower. However, if a task involved only walking^27–29^, conflicting results were obtained in terms of walking speed. Lee et al.^29^, for instance, found the glaucoma group to be slower, while Gomes et al.^28^ and Mihailovic et al.^27^ did not find an effect of glaucoma on walking speed. In our study, we found that GLA completed paths slower than C by 8.2 ± 2.3 s (39%). Our results match studies that involve demanding tasks during walking^24–26^, due to the nature of the path integration paradigm, which needs cognitive effort. Moreover, we observed that GLA gave slower responses in pointing task by 5.3 ± 2.1 s (46%), which was similar to a previous wayfinding study^19^. We also found that travel- and pointing-time to be related to the size of visual field defects, similar to previous studies demonstrating increase duration in both wayfinding^19^ and walking tasks^27^. Remarkably, travel-time duration correlated inversely with pRNFL thickness. This is of particular interest, as this OCT-based measure is an objective marker of glaucoma damage within the retina. Our finding therefore, appears to offer an intriguing link between retinal damage and higher-level behavior that deserves attention in further research.

### Effect of lighting on distance error

We did not find a group effect in distance error, but there was a lighting condition effect which might be associated with age. Accuracy was higher in daylight condition (by 12%), while travel- and pointing-time duration were slower in daylight condition (by 4% and 27%), respectively. Therefore, we suggest that participants might have traded off time for accuracy in daylight. It should be noted that the effect is lost when we consider age as a covariate and might hence be a signature of aging^30,31^.

### Practical considerations of VR-testing in eye diseases

VR-based path-integration paradigms in glaucoma appear to open a number of opportunities. The paradigm covers not only the mobility-related features, as reported previously^12,13^, but also cognition-related features of navigation as reported in the present study. Therefore, path-integration paradigms might be of promise to understand the relation of disease progression and higher cognitive tasks such as navigation. While in healthy participants performance appears to relate less on travel duration than to travel distance^32^, we suggest that in glaucoma duration measures in the path-integration paradigm could serve as biomarkers. Finally, VR tools enable us to manipulate environments in systematic ways, as in the present study where we compared the effect of different lighting conditions. Upcoming experiments might take advantage of this option, e.g. by studying the effect of simulated visual field defects on performance.

### Limitations

In the present study, there are several limitations that might deserve consideration. One limitation is that we only tested central vision: The participants could only use the central visual field to complete the task in VR via the HMD, i.e. HTC Vive Pro^33^, where the participants’ visual fields covered 47° and 40° temporal and nasal from fixation, respectively. In order to understand the effect of peripheral visual loss in path integration, an HMD with bigger field of view to show the stimuli might be of benefit. However, due to current stage of the VR technology, there has not been any HMD developed to fully cover the peripheral visual field^33^. Another limitation is related to the difficulty of luminance calibration for the two lighting conditions (daylight vs dawn) for HMDs. Further, it should be noted that VR-setups bear a risk of cybersickness in general, but less likely in walking-based techniques in VR^34^. Finally, in the present proof-of-concept study, the cohorts were small (GLA = 14, C = 15) due to restrictive inclusion criteria. Moreover, our GLA included mainly early and few advanced glaucoma cases.

## Conclusion

We assessed the navigation performance of glaucoma using path-integration paradigm in VR considering both cognitive-based and mobility-based skills employed in navigation. We found that our results correspond to previous navigation studies in real life and extended the knowledge by uncovering a relationship to objective, i.e. OCT, measures of glaucoma progression. Therefore, applying VR tools to study navigation performance in greater sample of glaucoma participant in simulated real-life environments offer a promising opportunity understand the relationship between the glaucoma progression and cognitive task performance.

## Methods

This observational study was conducted in a collaboration of the Ophthalmic Department of Otto-von-Guericke University of Magdeburg and the German Center for Neurogenerative Diseases (DZNE), Magdeburg, after the approval of the local ethical committee of the Otto-von-Guericke University of Magdeburg, Germany adhering to Declaration of Helsinki guidelines. Study participants gave written informed consent.

### Participants

15 (8 females) normally-sighted controls (C) and 15 (7 females) glaucoma patients (GLA) were initially recruited for the study. One glaucoma patient dropped out due to discomfort with the virtual reality equipment, resulting in 14 patients. Both groups cover a similar age range in years (mean and range for C vs GLA: 55.4 [42–78] vs 63.3 [51–80]), $p>0.05$ (t-test). All control and glaucoma participants underwent complete ophthalmological characterization at the Ophthalmic Department, including testing best corrected visual acuity (BCVA), visual field sensitivity (VF [MD]), optical coherence tomography (OCT) and cognitive tests. Subsequently, both groups participated in path integration task using an immersive virtual reality setup at the DZNE.

### Ophthalmologic characterization

BCVA was assessed using charts of early treatment of diabetic retinopathy study (ETDRS) at 4 m. Visual field sensitivity (VF) was investigated with the Humphrey Field Analyzer 3 using the Swedish Interactive Threshold Algorithm with 24-2 protocol (SITA-Fast, Jena, Germany). The sensitivity is given as mean deviation (MD), which is the difference in sensitivity compared to a matched reference population. For structural retinal readouts, peripapillary retinal nerve fiber layer (pRNFL) thickness was measured using OCT “Spectralis Glaucoma Module” (Heidelberg Engineering, Heidelberg, Germany); in one glaucoma patient the pRNFL thickness was measured with the regular OCT circular scan only. All OCT measurements satisfied the quality check, which were signal-to-noise-ratio > 15 dB.

According to Hodapp-Perish-Anderson criteria^35^ three glaucoma patients were classified in advanced stages and two of them were in mild stages while the rest were pre-perimetric when considering their better eyes in terms of visual field defects. The characteristics of participants are summarized in supplementary table 1.

### Inclusion and exclusion criteria

Inclusion criteria included for C: i) BCVA ≤ 0.1 logMAR and ii) normal VF (MD > − 4 dB); for GLA, as detailed in our previous study^36^: (i) open angle (OAG) with open anterior chamber, (ii) glaucomatous VF defects, and (iii) glaucomatous optic disc abnormality, e.g., cup-disc ratio ≥ 0.7, neuroretinal rim notching. Exclusion criteria included any systemic diseases restricting mobility of participants, e.g., mild cognitive impairment, and/or other ocular diseases affecting visual function.

### Cognitive and mobility assessment

Since the path-integration task requires cognitive abilities, cognitive function was assessed with the German version of the standard Montreal Cognitive Assessment Test (MoCA)^37^. Participants in both groups reached the score 24 or more out of 30, i.e. sufficient cognitive abilities to complete the task^38^. The activity of participants in their last four weeks was determined with the Life Space Assessment (LSA)^39^ test that allows for the assessment of mobility in different aspects of life such as physical and daily activities. In terms of LSA score, C ($\mu =86.5$, $\sigma =17.4$) and GLA ($\mu =79$, $\sigma =20.1$) groups did not have any significant difference ($p=0.29;$ t-test). Overall, the two groups did not differ in terms of cognitive skills and mobility.

### Path-integration task

#### VR task and setup

Path integration task was conducted in a virtual environment. The environment was created and the paradigm was implemented using a game engine (Unity v2019.2.0f., Unity Technologies, CA, USA). The virtual area of the experiment was of 4 m × 6 m, which was sufficient to perform the experiment without problems and limitations. Participants wore a head mounted display (HMD), HTC Vive Pro (HTC Cooperation, Xindian, Taiwan) and held a motion-tracked controller to interact within the environment while wearing their refractive corrections.

The path integration task consists of two main sub-tasks (i) travel task and (ii) pointing task with a total of three outcome measures as detailed below (a–c). (i) Travel task: The participants walked towards three different checkpoints, appearing one after the other and each disappearing upon the participant’s visit. (ii) Pointing task: Once the participants reached the last checkpoint, they physically rotated to face the initial checkpoint to estimate the direction and used the controller to indicate the distance, as detailed below.

#### VR testing procedure

##### Learning Phase

The main aim of the learning phase was to help participants to familiarize with the virtual experimental procedure and the indication of their distance estimates so that we reduced the adaptation effect on the actual experiment. The learning phase consisted of **Phase I** ‘distance judgement task’ (not included in the actual experiment), **Phase II** ‘travel task’, and **Phase III** ‘pointing task’.

**Fig. 4:**
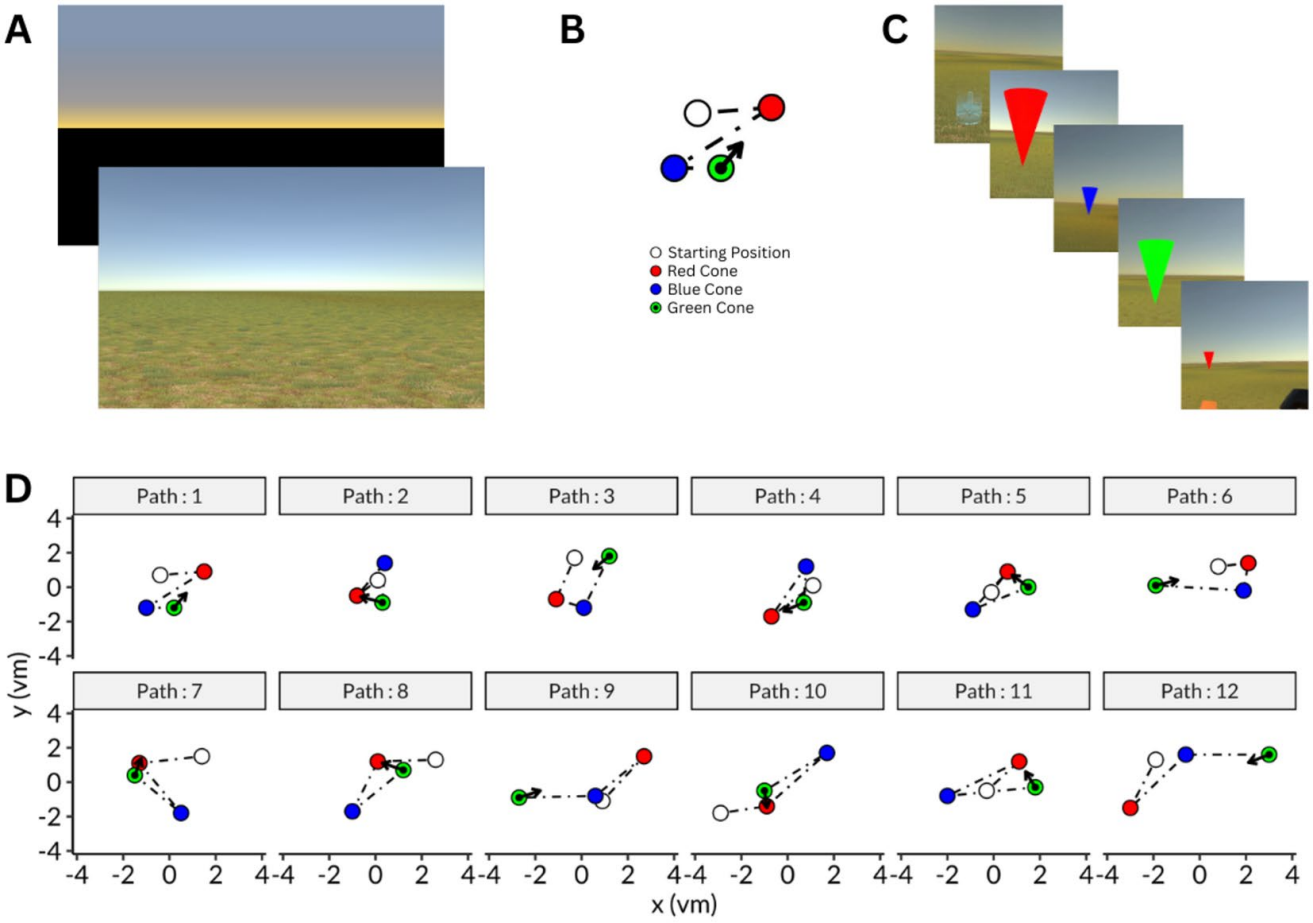
Path integration task. (**A**) Environments used in the paradigm to represent dawn (top) and daylight (bottom) conditions. (**B**) An example of a path shown with checkpoints and the expected direction response. Participants followed a trajectory with checkpoints as indicated by the colored points: white point is the starting point; the red one represents the first checkpoint that is to be remembered; for the yellow point, participants are instructed to point back to the red point. (**C**) First person view of each event in VR shown in B for daylight condition from top-left to bottom-right. At the last event (bottom-right), participants used the remote controller and their body to adjust the position and the size, i.e. distance, of the cone in order to indicate the first checkpoint. (**D**) Scheme of the 12 distinct paths applied (combination of 4 different path lengths and 3 different angles); arrows indicate direction of first checkpoint.

**Phase I**. The distance judgement task was performed independently from other two tasks. This consisted of the following: (a) a cone was presented at a random VR distance and disappeared after 5 s, (b) the participant did a full body turn, (c) an object was presented at a random location, (c) the participant positioned the presented object to the presumed location of the original cone position. The object presented last was cuboid, different from the cone-shape to allow distance estimation based on perception rather than pattern matching strategies of objects. This task was repeated 3 times for two lighting conditions, daylight and dawn (Fig. 4A). In daylight condition, the virtual scene had a terrain with grass texture and a lighting source while in dawn condition, the lighting source was removed and the terrain was covered with black. Physical luminance could not be determined due to the presentation with VR-goggles. Throughout the training, participants became faster at responding. In the first training trial, their response time ranged from 22.5 to 120.8 s (mean: 49.6 s). By the end of the training, their response time decreased to a range of 11.1 to 34.4 s (mean: 18.8 s).

For Phases II and III see below (Experiment), participants completed on two different paths in order to check whether they had correctly followed the instructions. If requested by the participants, the learning phase was repeated for more clarity. In the training phase, participants completed Phase II within 23.7 s to 112.6 s (mean: 46.8 s) and Phase III within 11.0 s to 50.3 s (mean: 27.0 s).

##### Experiment

The main experiment was divided into four blocks, i.e. ‘daylight’, ‘dawn’, ‘daylight’, ‘dawn’ (scheme: ABAB), each comprising 12 distinct triangular trials (Fig. 4D). The paths were generated for four different path lengths [4.5, 5.4, 6.4, and 7.5 virtual meters (vm)] and three different angles [58°, 108°, and 158°], which are the minimum angles for participants to align themselves on the line defined in Euclidian space which intersects with the first and the final checkpoints. Path order was randomized upon block repetition.

In each trial, the participants started at the initial location, indicated by an arrow, to begin navigating physically through three sequential checkpoints, i.e., marked by red (first), blue (second), and green (last) cones, which were clearly visible to ease completion the trial even with visual impairment (Fig. 4C). Between these checkpoints, a pointing arrow was presented to guide the participants to the next destination. At the final checkpoint, green cone, the participants were instructed to face the position of the first checkpoint at which the red cone had been located, only by turning their bodies. Then, they used the controller to adjust the distance (Fig. 4B). The next trial started right after their response.

The details of the outcome measures are defined as follows: Travel-time (i) is the time that takes for a participant to complete a trajectory by walking in one trial, excluding the time required for the participant to find the next checkpoint. The excluded time was defined by the time spent in the area within 10 cm from the current checkpoint. Pointing-time (ii) is the duration starting from the time of reaching the final checkpoint and ending at the time at which the participant gave the response by pointing at the first checkpoint. Euclidian-distance error (iii) is the Euclidian distance between the first checkpoint and the response location.

### Analysis and statistics

Analysis of the data was performed with R^40^. Normality checks were performed with Shapiro–Wilk test. The average of the two trials in each path under respective lighting conditions was calculated. For each outcome measure, repeated measure mixed ANOVA was conducted to identify the main effects of GROUP (between subject variable), LIGHTING condition (within subject variable), and their interaction.

We also explored the relationship between outcome VR measures and ophthalmological characteristics (VF [MD], pRNFL, and BCVA) from the better seeing eye using linear regression with bisquare weighting^23^, which is robust to outliers in an analysis.

## Supplementary Information

Supplementary Table 1.

## Data Availability

Data are available on request. Please contact the corresponding author for the data availability.
