# Supplementary material for: Navigation performance in glaucoma: virtual-reality-based assessment of path integration
Source: Sci Rep. 2024 Sep 12;14:21320. doi: 10.1038/s41598-024-72040-8 (PMC11393326; doi:10.1038/s41598-024-72040-8)
Supplement: Supplementary file 1 — Supplementary Table 1. [file 41598_2024_72040_MOESM1_ESM.pdf]

Supplementary Materials

Participant	Age[y]	Sex	Visual Acuity Decimal		Visual Acuity logMAR		Visual Field MD [dB]		pRNFL [µm]	
			OD	OS	OD	OS	OD	OS	OD	OS
**Control**										
C01	42	f	1.60	2.00	−0.2	−0.3	0.07	0.67	97	102
C02	59	m	2.00	2.00	−0.3	−0.3	0.62	-0.08	100	98
C03	42	f	2.00	2.00	−0.3	−0.3	0.12	0.27	104	107
C04	49	m	1.25	1.00	−0.1	0.0	-0.70	-0.78	88	93
C05	66	f	1.00	1.25	0.0	−0.1	-0.90	-0.48	91	95
C06	46	f	1.00	1.25	0.0	−0.1	0.74	0.49	105	100
C07	44	f	1.25	1.25	−0.1	−0.1	-0.37	-1.08	98	93
C08	46	m	1.60	1.60	−0.2	−0.2	0.10	0.64	92	92
C09	64	m	1.60	1.60	−0.2	−0.2	0.81	-0.04	82	83
C10	53	m	1.60	1.60	−0.2	−0.2	1.09	0.75	106	109
C11	42	f	1.25	1.25	−0.1	−0.1	0.43	0.33	95	96
C12	63	f	1.25	1.25	−0.1	−0.1	-0.03	-0.47	111	114
C13	64	m	1.25	1.60	−0.1	−0.2	1.18	2.05	93	94
C14	73	m	1.00	1.25	0.0	−0.1	-0.05	-0.92	84	92
C15	78	f	1.00	1.25	0.0	−0.1	0.59	-0.58	94	76
**Glaucoma**										
G01	56	m	NA	0.63	NA	0.2	NA	-31.22	NA	37
G02	64	m	NA	NA	NA	NA	-29.99	NA	56	NA
G03	51	f	0.03	NA	1.6	NA	-32.56	NA	28	NA
G04	53	m	1.25	1.25	−0.1	−0.1	0.12	-9.63	98	74
G05	68	m	1.00	0.80	0.0	0.1	-7.19	-0.44	79	80
G06	61	f	1.25	1.25	−0.1	−0.1	-1.55	-3.25	94	97
G07	60	m	1.60	1.25	−0.2	−0.1	-1.38	-3.38	89	78
G08	62	f	1.25	1.25	−0.1	−0.1	0.93	0.27	96	94
G09	74	f	0.63	0.80	0.2	0.1	-2.98	-5.00	84	77
G10	66	m	1.60	1.25	−0.2	−0.1	-0.45	-1.18	78	66
G11	80	f	0.63	0.80	0.2	0.1	-17.86	-3.51	57	80
G12	52	f	1.00	1.00	0.0	0.0	-1.88	-1.22	106	105
G13	60	f	1.25	1.00	−0.1	0.0	-0.33	-6.44	83	75
G14	79	m	0.32	0.63	0.5	0.2	-0.10	-16.12	80	44

Age[y], *Age in Years;* ***OD***, *Right Eye;* ***OS***, *Left Eye*
MD, *Mean Deviation;* ***pRNFL***, *Peripapillary Retinal Nerve Fiber Layer*
f, *female;* ***m***, *male;* ***NA***, *Not Available*

Supplementary Table 1. Summary of participant visual characteristics
